# Moving Myeloid Leukemia Drug Discovery Into the Third Dimension

**DOI:** 10.3389/fped.2019.00314

**Published:** 2019-07-30

**Authors:** Donna M. Cartledge Wolf, Sigrid A. Langhans

**Affiliations:** Nemours Center for Childhood Cancer Research, Nemours/Alfred I. duPont Hospital for Children, Wilmington, DE, United States

**Keywords:** three-dimensional cell culture, bone marrow, leukemia, AML, tumor microenvironment

## Abstract

The development of therapies aimed at leukemia has progressed substantially in the past years but childhood acute myeloid leukemia (AML) remains one of the most challenging cancers to treat. Genomic profiling of AML has greatly enhanced our understanding of the genetic and epigenetic landscape of this high-risk leukemia. With it comes the opportunity to develop targeted therapies that are expected to be more effective and less toxic than current treatment regimens. Nevertheless, often overlooked in leukemia drug discovery are the dynamic interactions between leukemic cells and the bone marrow environment. The interplay between leukemic cells, stromal cells and the extracellular matrix plays critical roles in the development, progression and relapse of AML as well as in drug response and the development of resistance. Here we will review pediatric leukemia with a special focus on acute myeloid disease in children, and discuss the tumor microenvironment in the context of drug resistance and leukemia stem cell survival. We will emphasize how three-dimensional (3D) cell-based drug discovery may offer hope for both the identification and advancement of more effective treatment options for patients suffering from this devastating disease.

## Introduction

Leukemia encompasses a set of malignant conditions that affect blood and blood forming tissues. Normal blood cells are derived from hematopoietic progenitor cells, which go on to differentiate into cells of either myeloid or lymphoid lineage. Myeloid cells become erythrocytes, platelets, myeloblasts, and granulocytes, while lymphoid cells develop into lymphoblasts that subsequently differentiate into B-lymphocytes, T-lymphocytes or natural killer cells (1, 2). In leukemia, normal hematopoiesis is suppressed by the uncontrolled proliferation and accumulation of leukemic cells (3). Leukemias are classified by the types of cells affected and are further defined by the developmental stage of the originating cells. They are grouped into four major categories: acute myeloid (AML) and acute lymphoblastic leukemia (ALL), and chronic myeloid (CML), and chronic lymphoblastic leukemia (CLL). Myeloid leukemias are derived from myeloblasts and lymphoblastic leukemias are derived from lymphoblasts, acute conditions arise from early, immature cells and chronic conditions are derived from mature, abnormal cells. Leukemia is generally considered an uncommon condition. In the United States in 2019, there were estimated to be approximately 1.76 million new cases of cancer diagnosed in the overall population with 61,780 being leukemia (4). Approximately 9% of newly diagnosed cases of leukemia are in children and young adults. Acute leukemias are the most common type and are responsible for approximately one third of all cancers in this age group. Mixed lineage leukemias (MLL) are a subtype of acute disease and have features of both AML and ALL. Chronic leukemias are very rare in children.

Identification of early drugs used to treat leukemia was not target driven. These compounds were, instead, already in use for various other diseases and disorders and were administered to patients in the hope of providing a path to recovery. Arsenic was the first recognized leukemia therapy and was a principal component in a potassium bicarbonate based solution of arsenic trioxide, developed in the late Eighteenth century by Thomas Fowler (5) and first used as a leukemia treatment in 1865 (6). Arsenic containing compounds, with or without concomitant radiation, remained the standard therapy for leukemia up until the introduction of busulphan in 1953 (6). L-asparaginase, an enzyme that catalyzes the conversion of L-asparagine to L-aspartic acid and ammonia, has been used to treat pediatric patients with ALL since the mid-1960s (7–9). In the first account of clinical use, Dolowy et al. showed that an 8 year-old child achieved a partial response upon treatment with this enzyme (10). Hill et al., subsequently reported that patients treated with L-asparaginase had significant improvement, even with advanced disease, and achieved complete remission in one of three patients tested (11). Clinical trials followed, which further demonstrated the efficacy of such a targeted treatment as both a stand-alone therapy (12) and in combination with other pharmaceutical agents (13). But it was not until recently that it was found that a cytogenetic and molecular subgroup of AML characterized by chromosome 7 monosomy could also benefit from L-asparaginase treatment (14), a treatment that may preferentially target leukemia stem cells in the bone marrow microenvironment (15).

In addition to the development of new drug treatment strategies, radiation therapy became part of the conventional methods used to treat leukemic disease (16). Since then, treatment options for leukemia have evolved, from technological advances such as stem cell transplants, to the development of new targeted therapies based on the increased understanding of molecular events leading to leukemia, to taking advantage of a patient's own immune system in immunotherapy. In this review, we will give an overview of disease subtypes, etiology and current treatment options. We will discuss the more recent understanding of the influence of the tumor microenvironment within the bone marrow on cancer stem cell proliferation and its impact on drug resistance. Lastly, we will address the challenges this poses for traditional drug discovery efforts and how new phenotypic 3D screening methods that can more closely mimic such a tumor microenvironment may help to overcome these limitations.

## Etiology of Myeloid Leukemias

Leukemias are thought to arise from a single mutant cell, but the cancer populations are not clonal. Several mutations are possible within the spectrum of an individual patient's disease. In accordance with Knudson's two-hit hypothesis (17), leukemias are cancers that are generally caused by two oncogenic events. Knudson's hypothesis, although ground-breaking for its time, does have some limitations because it is now known that several tumor suppressor genes (TSGs) do not fit within its confines. In an effort to expand and redefine this paradigm, Paige placed these TSGs into three main categories, including those that arise from (a) monoallelic disruption (haploinsufficiency, dominant negative and gain-of function isoforms); (b) multiple gene interactions (multi-step tumorigenesis, genetic modifiers or mutators); and (c) dual function TSGs that have both tumor-suppressing and tumor-promoting properties [reviewed in (18)]. Oncogenic events include deviant expression of proto-oncogenes, and chromosomal abnormalities that result in changes in chromosome number, chromosome inversions, or creation of translocations leading to gene fusions and subsequent modification(s) of cell signaling pathways due to over or under activity of kinases and/or transcription factors (3, 19, 20). A subset of AML, referred to as t- or therapy related-AML are, as the name suggests, caused by mutations that arise as a result of treatments for other disease conditions, including benign and malignant neoplasms and immune system disorders. Prior disease management may include individual or combined treatments including chemotherapy, radiation, and immunosuppressive therapies (21, 22). Prognosis for patients with t-AML is generally very poor; supportive therapy is most often the only treatment option (23).

Irregularities at the *Mixed Lineage Leukemia* gene (*MLL*) locus present on 11q23 are frequently responsible for aggressive cancers that most often occur in the pediatric population and are due to conversion of *MLL* into an active oncogenic state (24). MLL has been shown to fuse with many partners including the Acute Lymphoblastic Leukemia 1-Fused Gene from Chromosome 4 (AF4), Acute Lymphoblastic Leukemia 1-Fused Gene from Chromosome 6 (AF6), Acute Lymphoblastic Leukemia 1-Fused Gene from Chromosome 9 (AF9), Acute Lymphoblastic Leukemia-1 Fused Gene from Chromosome 10 (AF10), Eleven-Nineteen-Leukemia (ENL), Eleven-Nineteen Lysine-rich Leukemia (ELL), CREB [cAMP (cyclic adenosine monophosphate) Response Element Binding] Binding Protein (CBP), Protein 300 (P300), ALL 1 [Acute Lymphoblastic Leukemia 1]-Fused Gene from Chromosome 1 Protein (AF1p), Growth Arrest Specific Protein 7 (GAS7), Abl-Interactor 1 (ABI1), and Extra Eleven-Nineteen Leukemia (EEN) proteins [reviewed in (24)].

Alcalay et al. showed that some fusion proteins in AML induced a mutator phenotype, down regulating the activity of DNA base excision repair genes (25). Hence, the presence of fusion proteins impaired DNA repair mechanisms leading to further DNA damage and induction of a leukemic phenotype. The NUP98-NSD1 fusion protein occurs in 4.4% of pediatric AML and is associated with a <10% event-free 4-year survival rate (26). NUP98, or Nucleoporin 98-kDa, is located on chromosome 11p15, and is part of the nuclear pore complex, which controls movement of protein and RNA between the nucleus and the cytoplasm (27). Chromosomal rearrangements are responsible for fusion of NUP98 with several different partner genes which may, broadly, be grouped into three categories: homeodomain; nuclear nonhomeotic, which includes NSD1; and cytoplasmic [reviewed in (28)]; (29). NSD1, or Nuclear Receptor-binding SET [Su(var)3-9, Enhancer-of-zeste and Trithorax] Domain Protein 1, is located on chromosome 5q35 and is a histone methyltransferase (30). NSD1 predominantly dimethylates lysine 36, located close to the globular domain of nucleosomal histone H3 (31). NSD1 retains methyltransferase activity in the fusion, and it is this property that is essential for leukemia progression (32). Aberrant expression of NUP98-NSD1 promotes leukemogenesis by activating transcription of hematopoietic regulatory genes, principally *Homeobox A* (*HOXA*), *Homeobox B* (*HOXB*), and *Myeloid Ecotropic Viral Integration Site 1 Homolog* (*MEIS1*), which subsequently activate down-stream proto-oncogenic target gene *Myeloblastosis Viral Oncogene Homolog* (*c-Myb*) (33, 34). *HOX* expression is markedly reduced as myeloblasts differentiate into mature hematopoietic cells (24). When *HOX* expression is continually stimulated, myeloblastic cells become self-renewing and fail to differentiate, thus exhibiting a stem cell-like, immortal phenotype (24) that most often leads to cancer.

Secondary events leading to leukemogenesis include activating mutations in additional proto-oncogenes such as the NOTCH1 transmembrane receptor, implicated specifically in T-cell derived ALL (35, 36). Mutations in the receptor tyrosine kinase FMS [Feline McDonough Sarcoma] Related Tyrosine Kinase 3, FLT3] (37) are often due to internal tandem duplications, referred to as FLT3 ITD (38) or due to point mutations in the tyrosine kinase domain in the codon for an aspartate (D835) or an isoleucine (I836) residue, collectively termed FLT3 TKD (39). Loss-of-function mutations in tumor-suppressor genes such as Retinoblastoma protein, pRb, and p53 have been described as well (40) and mutations in non-coding regions of DNA have also been implicated in malignant transformation (41).

## Current Treatment Options

Treatment for leukemia generally involves a series of two to three steps: (a) induction therapy which is intended to bring the patient into remission, (b) consolidation therapy, designed to eradicate cancer cells that may have escaped front line treatment strategies, and (c) maintenance therapy, with the goal of keeping the patient in a disease remissive state. Clinically, remission is defined as a significant decrease in detectable disease, and is most often concurrent with a considerable reduction in symptomatology. With respect to cancer patients, total remission entails the inability to detect cancer in the body with current diagnostic technologies. Although this outcome is certainly encouraging, it does not mean that a patient is cured. Ideally, treatment would be both innocuous and lifelong, such that any signs of recurrence would be promptly addressed and eradicated.

Currently, for the pediatric population, management of AML involves predominantly induction and consolidation therapies. Whereas, >80% of children diagnosed with AML will achieve remission, only about half will remain disease-free for an appreciable period of time. Children who fail treatment are often referred for hematopoietic stem cell transplantation (HSCT), which can be administered after the first complete remission or subsequently, following one or more relapses of the disease [reviewed in (42)]. Biomarker guided treatments are evolving, with molecular targets including cell surface antigens, disease-associated regions of proteins, and enzymes. While treatment options vary according to risk group, preliminary therapy with cytarabine in combination with anthracycline drugs such as topoisomerase inhibitors is favored, and is often combined with purine antagonists and sometimes combined with cytokine exposure (43, 44). The cytotoxic antitumor drug-conjugated antibody gemtuzumab ozogamicin has been approved by the US Food and Drug Administration (FDA) for use in children 2 years of age and older with CD33-positive AML, but not as a first line treatment strategy. This drug is only indicated for use in children who have failed traditional treatment by either exhibiting no response or by having a recurrence of their disease. The antibody portion of the conjugate binds to Cluster of Differentiation 33 (CD33), a cell surface adhesion protein that is present on leukemic blasts and immature normal myelomonocytic lineage derived cells, but not on normal hematopoietic stem cells or on non-hematopoietic cells. After attachment to CD33, the drug-linked antibody enters the cell via endocytosis and the drug is released in the lysosomes after which it binds to DNA and causes double-stranded DNA breaks, leading to cell cycle arrest and ultimately to cell death by apoptosis (45, 46).

Chimeric antigen receptor (CAR) T-cell therapy is a type of personalized medicine in which the patient's own immune system is primed to destroy malignant cells. CAR T-cell therapy has, so far been approved by the FDA for the treatment of patients from 3 to 25 years of age with B-cell derived ALL who have failed standard treatments by either having no response or who have responded, achieved remission and then have had their cancer recur two or more times (47, 48). This therapy, termed “Tisagenlecleucel,” involves (a) collection and purification of T-cells from the patient's body; (b) engineering of these cells to express CAR; and (c) transfusion of these cells back into the patient (49). While these T-cells are being genetically altered in a process that takes about one month's time, the patient receives lymphocyte depleting chemotherapy so that when reintroduced, these engineered cells stand a better chance of recognizing and eradicating the cancer (49). Efficacy of this treatment relies on the ability of CAR expressing T-cells to recognize Cluster of Differentiation 19 (CD19), a cell surface antigen that is highly expressed on leukemic B-cells, but insignificantly present on normal B-lymphocytes (50). While CAR T-cell therapy is still in its infancy, with our steadily increasing understanding of molecular events leading to the development and progression of AML, it will only be a matter of time before CAR T-cell therapy will also become a treatment option for pediatric AML patients.

Treatments that combine chemical or cell destabilizing agents with physical disruption include photodynamic (PDT) and sonodynamic (SDT) therapies. The intent of these treatments is targeting and damaging malignant cells. As their names suggest, the energy sources for PDT and SDT are light and sound, respectively. Although both light and sound waves can be focused, sound is advantageous over light because it can penetrate deeper into the body and can be effectively aimed at hard to reach or otherwise inoperable tumors. SDT, extensively reviewed in (51), relies on administration of a sonosensitizing agent and exposure to ultrasound irradiation that is focused on the tumor. These sound waves disrupt the sensitizing compound, causing it to transform into an excited state, which then reacts with molecular oxygen and generates reactive oxygen species (ROS), which go on to disrupt mitochondria and ultimately leads to apoptosis (51). Although PDT and SDT are emerging therapies for various types of cancer, each treatment modality is applicable for eradication of leukemic cells of both myeloid and lymphoid origins.

**Figure 1:**
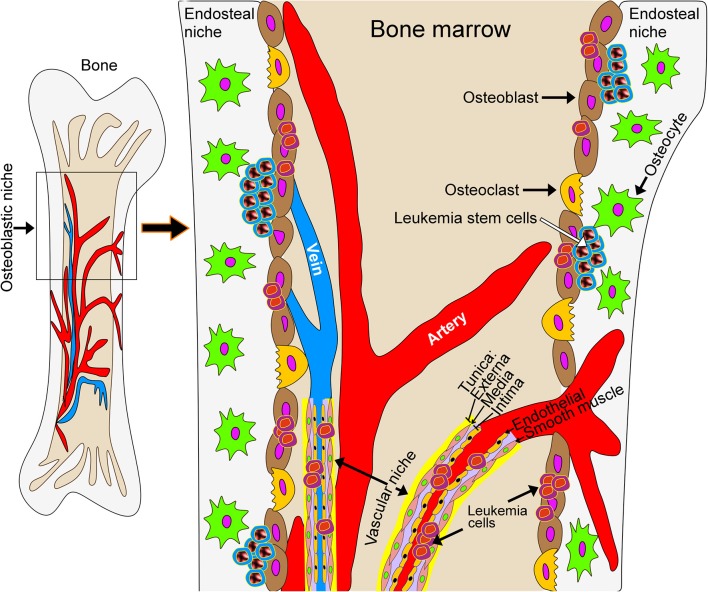
Schematic model of the leukemic bone marrow niche. The microenvironment in the leukemic bone marrow niche contributes to disease progression, therapeutic response, evasion of therapy and the development of resistance to treatment. New treatment strategies may target the interaction of leukemic cells with stromal cells or with the extracellular matrix underlying bone tissue.

Development of therapies aimed at leukemia has progressed substantially in the last decade. Pediatric disease targets, extensively reviewed in (52), include protein tyrosine kinases, protein serine/threonine kinases, proteases, anti-apoptotic proteins, DNA methyltransferases, histone deacetylases, and chemokine receptors. The National Cancer Institute (NCI) online database lists over 350 clinical trials for various types of leukemia (see Supplementary Information). Most allow pediatric patients, defined here as being <18 years of age. This population is, however, generally under-represented. The Children's Oncology Group (COG) currently has 18 clinical trials addressing various hematologic malignancies in the pediatric population. Still, most experimental therapeutics target intrinsic properties of leukemic cells. However, and in particular in myeloid diseases, there is increasing evidence that the microenvironment in the leukemic bone marrow niche contributes to disease progression, therapeutic response and evasion of therapy as well as the development of resistance to treatment (Figure 1). While this opens up avenues for the development of new therapeutic strategies that target the tumor microenvironment, current phenotypic screens using leukemic cells in suspension culture are poorly suited for drug discovery approaches targeting the interaction between leukemic cells and the tumor microenvironment.

## AML and the Bone Microenvironment—a Chance for Developing New Therapeutic Strategies

AML stem cells reside in the bone marrow niche and are generally refractory to chemotherapy, necessitating the development of new molecularly directed therapeutics. The question of AML stem cell origin is complex at best. The idea of a specific region in the bone giving rise to and influencing the maturation and ultimate fate of hematopoietic stem cells, in the normal non-malignant context, was first suggested by Schofield who, in 1978, referred to these areas as “stem cell niches” (53). There are currently two theories on this phenomenon. The first being that chemotherapy causes genetic and epigenetic changes in AML cells that lead to the emergence of those with drug resistance. The second stating that these refractory stem cells are already present (before treatment) and that chemotherapy simply kills off susceptible cells, effectively selecting for those with endogenous resistance (54). Chemotherapy does certainly select for resistant cells in the clonal population, but within the context of AML, stem cells are an important factor in both disease persistence and recurrence.

In recent years, it has become clear that leukemic cells influence the bone marrow microenvironment and, at the same time, the bone marrow microenvironment influences leukemic cells. This becomes a problem when the interactions with the bone marrow leukemic niche promote the evasion of leukemic cells from treatment. For example, the bone marrow microenvironment contains a wealth of components that safeguard leukemia cells from treatment-induced demise. Cytotoxic insult leads to drug resistance by way of one or more environmental methods, classified as soluble factor- and cell adhesion-mediated mechanisms [reviewed in (55)]. Secretion of cytokines, especially those of the Cysteine-X-Cysteine chemokine ligand (CXCL) structural motif, in the bone marrow microenvironment attracts Cysteine-X-Cysteine chemokine receptor (CXCR)-bearing leukemia cells in the bone marrow. The CXCL12/CXCR4 ligand/receptor axis is associated with AML, and patients whose cancers over express CXCR4 tend to have aggressive disease and a poor overall prognosis (56–58). On the other hand, AML cells modify the bone marrow microenvironment by producing angiogenic factors that restructure vascularity within endosteal niches and promote a phenotype that favors AML growth but hinders propagation of normal hematopoietic stem cells (HSC) (59). This results in loss of normal HSC which plays a major role in leukemia progression. Duarte and colleagues showed that when the endosteal niche is protected, either by chemical or by genetic means, efficacy of chemotherapeutic treatments is substantially increased (59). Exosome secretion has recently been implicated in modifications observed in the marrow of AML permeated bone (60). Kumar and colleagues found that exosomes were significantly increased in the bloodstream of AML patients when compared to blood from normal controls, and that up regulation of Dickkopf-1 (DKK1) was positively correlated with the presence of these vesicles (60). DKK1 is secreted by mesenchymal stem cells, including osteoblasts, is involved in bone and blood cell formation, and is a potent inhibitor of Wingless-Int (Wnt) pathway signaling (61). In contrast to most reported findings (62–64), this discovery suggests that reduced Wnt signaling may also play a role in AML-driven bone marrow remodeling. An ideal strategy for circumventing such problems will be to identify inhibitors that disrupt the interplay between AML and the bone marrow microenvironment. However, genetic alterations are not limited to leukemic cells but molecular alterations can also be found in bone marrow mesenchymal stromal cells of AML patients opening up the possibility of niche-directed therapies in AML (65).

Like all cancers, AML becomes fatal when patients fail to respond to therapy or when they respond initially then, after a period of time, experience recurrence with cells that have become resistant to intervention. Despite subsequent administration of dissimilar therapeutics, the phenomenon of acquired resistance often replays itself numerous times until no effective disease management strategies remain. Preventing relapse is the key to keeping the cancer at bay in a state that is compatible with life, i.e., stable disease, or to eradicating the disease completely. Since growth within the confines of the bone marrow microenvironment and/or growth in multicellular clusters afford protection to AML cells, a way to prevent these hidden cells from surviving is to identify drugs capable of reaching and destroying them. Cytotoxicity of therapeutics targeting AML cells that are rapidly dividing will fall short in eradicating those in a quiescent state, and is of particular concern with regard to leukemia stem cells (66). In both cases, a fair percentage of AML cells residing in the bone marrow niche are slowly or not actively growing. A way to discover these types of first-in-class therapeutics is to combine the best aspects of phenotypic and target-driven high-throughput screening (HTS) in drug discovery. Phenotypic because drug induced effects on cells are investigated, and target-driven in that cells are grown in a format that more closely replicates *in vivo* conditions. From this perspective, co-culturing AML cells with stromal cells is a good first step in mimicking these circumstances.

## A Case for Co-culturing AML and Stroma Cells

Phenotypic changes resulting from cell-cell interactions are factors that need to be considered in defining how the bone marrow niche contributes to AML survival. In a recent study, Zeng et al. used a mechanism-based selection strategy to identify combinations of drugs to eliminate bone marrow microenvironment-mediated resistance in AML (67). Interestingly, this method utilized co-culturing of human leukemic cells and mouse derived stromal cells under more traditional two-dimensional (2D) culture conditions. A 2D niche-based phenotypic screen utilizing T-ALL derived mouse leukemia stem cells co-cultured with mouse stromal cells genetically altered for optimal activation of the transmembrane receptor NOTCH1 identified compounds that were selectively toxic to stem cells of lower proliferative state (68). In this study, expression of the transcription factor c-Myelocytomatosis oncogene cellular homolog (c-Myc) was implicated as a causal factor for drug resistance in leukemia cells, specifically those of the stem cell state. NOTCH1 signaling promotes self-renewal and survival of hematopoietic progenitor cells (69), and has been shown to induce c-Myc expression and to augment cell growth in leukemia (70). In an effort to elucidate the mechanism by which interactions between AML and stromal cells increases c-Myc expression, Tian et al. performed microRNA array analysis on AML cells from patients and AML cell lines cultured with and without human stromal cells and found differences in the expression levels of various microRNAs under both growth conditions (71). MicroRNAs are small RNA molecules that do not code for protein, and function in gene silencing and in post-transcriptional modifications by binding to and interacting with the 3′-untranslated region (UTR) of target genes (72). When AML cells were grown in close proximity to stromal cells, expression of microRNA-494 was down regulated, allowing c-Myc expression to be maintained at enhanced levels which confers drug resistance to AML cells. In agreement with this finding, primary AML cells taken from patients whose microRNA-494 expression was weak were found to have worse prognoses than those whose levels were at normal or enhanced levels. In another study utilizing 2D co-culturing of AML and stromal cells, all of human origin, Xia et al. showed that expression of c-Myc was significantly up-regulated in primary AML cells from patients and AML cell lines grown in the presence of human mesenchymal stromal cells (73). Expression of c-Myc conferred resistance to apoptosis induced by the type II topoisomerase inhibitor, mitoxantrone, while inhibiting c-Myc either by expression (siRNA) or functional (small molecule inhibitor) based approaches abrogated mitoxantrone resistance in these cell lines. Interestingly, in order to be protected from mitoxantrone induced apoptosis, AML cells needed to have cell-to-cell contact with stromal cells. Exposure of these cells to stromal cells produced soluble factors that, in the absence of this contact, afforded no chemotherapeutic protection.

In another study, investigation of 53 fundamental proteins in 11 cell signaling pathways showed that AML cells responded differently to treatment with Mechanistic Target of Rapamycin (MTOR) inhibitor temsirolimus, B-cell lymphoma 2/B-cell lymphoma-extra large (BCL2/BCL-XL) antagonist ABT737, and mouse double minute 2 homolog (MDM2) inhibitor Nutlin-3a based on whether or not they were grown in the presence or absence of stromal tissue (67). While initial findings were obtained from human AML cells co-cultured with mouse stromal cells, stroma-mediated differences were also found in the presence of human stromal cells. Modified drug sensitivities were due to stromal-induced changes in AML signaling, and resulted in chemo-resistant phenotypes in most cases. Importantly, the authors found that simultaneously blocking phosphatidylinositol 3-kinase (PI3K)/protein kinase B (AKT)/MTOR and BCL2-associated cell signaling pathways was an effective means to deter stromal-mediated AML survival. This study supports the notion that directed therapeutics also have off-target effects on other proteins in other cell signaling pathways. As these stray targets have the potential to be therapeutically exploited, they are also clinically relevant.

## Disease Modeling of AML in Scaffold-Based Three-Dimensional (3D) Cultures

Leukemias are thought to persist during and after chemotherapeutic treatments due to regional proximity to and interactions with mesenchymal stromal cells of the bone marrow niche which (a) provide physical protection, and (b) bring about drug resistance in leukemia of both stem and blast designations. However, discovering drugs to circumvent this phenomenon should consider both the complex interactions of these cells with each other and also those with their extracellular environment. Growing cells, especially cancer, in 3D format is now considered to more closely resemble phenotypic characteristics of the originating tumor, namely cell morphology, proliferation potential, proliferation rate, and response to chemical, biologic, and radiation-based therapeutics [reviewed in (74–78)], thus providing a new means in drug discovery. As the bone marrow niche is itself a 3D structure, it makes sense that more closely replicating this phenotype would be an ideal method for identifying potential therapeutics, especially in phenotypic HTS. Phenotypic 3D cell culture may be applied to many facets of drug development including drug discovery through HTS, target identification and validation, drug characterization and toxicity profiling, and disease modeling to determine efficacy and safety of investigational new therapeutics (74, 78).

In the broadest sense, 3D cell culture may be broken down into two distinct categories—those that contain scaffolds and those that do not. Scaffold-bearing models support anchorage dependent growth, while scaffold-free systems enable growth with anchorage independence. Common models of 3D cell culture [reviewed in (78)] are scaffold-based biological and synthetic hydrogels, and scaffold-free hanging drop, low attachment microplate, and magnetic levitation methods. Replicating the *in vivo* environment of AML cells would best be served by co-culturing AML cells with mesenchymal stromal cells in a matrix-based scaffold, either of biological or synthetic origin. Advantages of biological scaffolding are that aside from the extracellular matrix proteins they contain sugars, amino acids, lipids, hormones and other soluble growth factors that are more reflective of the tumor microenvironment (79). These advantages have drawbacks, however, since natural products are fraught with innate differences leading to variability between lots and their composition depends on the tissue of origin. Synthetic scaffolds lack these intrinsic properties but are advantageous because of their reproducible uniformity. These structures consist of biologically compatible polymers and hydrogels (80, 81). Nutrients and growth factors can be added purposefully, which makes for a more chemically defined supportive growth structure. Because of their defined chemical composition and batch-to-batch consistency, synthetic scaffolds are expected to provide more reliable and reproducible results in drug screening.

In an effort to more closely mimic the AML tumor microenvironment, Houshmand et al. grew human TF-1 erythroblasts and human bone marrow mesenchymal stem cells together in a glass slide mounted microfluidic chamber in both two and three dimensions (82). For 2D, collagen was injected into the chamber. In 3D, demineralized bone matrix coated with collagen was loaded into the chamber. Cell culture medium was perfused throughout the system under both conditions. Phenotypic characteristics of TF-1 cells grown in 2D and 3D formats were investigated, and it was shown that cellular proliferation rates, percentage of cells in S, G2, and M phases, and resistance to chemotherapeutics was significantly increased in cells grown under 3D vs. 2D conditions (82), highlighting the relevance of 3D cell culture in leukemia drug discovery.

In a phenotypic study where human leukemic cell lines were co-cultured with human bone marrow-derived mesenchymal stem cells on a synthetic co-polymeric 3D scaffold, effects of chemotherapeutics on leukemic cells were investigated (83). Drug response of leukemia cells grown in 3D co-culture was compared to that of cells grown in 2D co-culture and in 2D suspension culture formats. Seeded scaffolds were shown to allow diffusion of molecules of up to 1,000 Da, including chemotherapeutics. Presence of stromal cells on scaffolding structures had a statistically significant, but realistically negligible difference on the drug absorption capabilities of these structures. Nevertheless, leukemia cells co-cultured with mesenchymal stem cells on 3D synthetic scaffolds had higher resistance to chemotherapeutics compared to cells co-cultured under 2D or singly cultured under 2D suspension culture formats. Immunohistochemical staining of cross sections of drug treated 3D co-culture models revealed decreased levels of Ki-67 protein, indicating reduced cellular proliferation that was both dose and time-dependent. Analysis of soluble factors collected from 2D and 3D co-cultures showed that these factors offered similar chemotherapeutic protection for cells grown under both conditions. Having produced equivalent results from two different culturing systems, soluble factors were not considered vital for stromal mediated chemotherapeutic protection of leukemic cells. The authors suggested that N-cadherin expression may play a role in 3D co-culture mediated chemoresistance in leukemia cells.

Along with the stromal niche, the vascular niche plays an important role in maintaining AML disease progression and chemoresistance. In a tri-culture model, human AML cells, human vascular endothelial cells and human mesenchymal stromal cells were grown together using star-shaped polyethylene glycol (starPEG)-heparin hydrogel scaffolds (84). To regulate growth conditions and to enable localized cellular remodeling, heparin components were covalently bound with cell specific adhesion ligands and growth factors, and hydrogel was permeated with matrix metalloproteinase-responsive peptides. Light and confocal microscopy revealed that after 1 week of tri-culture, most AML cells grew in mixtures of spheroids and clusters and accumulated along the vascular endothelial and mesenchymal stromal networks. AML cells were also grown in 2D and 3D co-culture with either vascular endothelial cells or mesenchymal stromal cells, and in 2D and 3D mono-culture formats. Resistance to chemotherapeutics daunorubicin and cytarabine was highest in AML cells grown under 3D tri-culture conditions. When, however, these two drugs were added in combination for a period of 5 days, viability of AML cells grown in 3D tri-culture was reduced by more than 90%. Since ~10% of the AML cell population survived this initial combination treatment, cells were grown in drug-free medium for 14 additional days. At the end of this recovery period, cell viability was not measurable and was thus, considered 0%, suggesting that a 3D cell culture system may serve more accurately as a model for drug resistance and combination therapy.

To replicate the hematopoietic stem cell bone marrow niche, a macroporous polyethylene glycol hydrogel was infused with a biologically active compound added to promote integrin receptor mediated cell attachment (RGDSK-PEG_6_-acrylate), co-seeded with human hematopoietic progenitor cells and human mesenchymal stem cells, and incorporated into a perfusion bioreactor system (85). It was thought that a perfusion system would be more representative of *in vivo* conditions, where nutrients, gases and secreted cellular factors are in continuous motion throughout the bone marrow. Flow rates are, however, adjustable in this system. In the dynamic setting, perfusion occurs. This represents activated conditions and promotes differentiation of hematopoietic stem cells. In static mode, perfusion does not take place. This corresponds to steady-state growth and favors maintenance of hematopoietic stem cells. When compared to treatment with DMSO vehicle control, hematopoietic progenitor cells exposed to chemotherapeutic compound 5-fluorouracil for 5 days under perfusion conditions had an ~22% death rate, while those under static conditions were more sensitive, dying at a rate of ~36% over this time period. Testing for the cell surface marker Cluster of Differentiation 34 (CD34), the presence of which identifies cells as being of hematopoietic progenitor subtype, indicated that CD34 positive cells were significantly less sensitive to 5-fluorouracil under static conditions. Under dynamic growth conditions, chemo sensitivity of CD34 positive and negative cell lines was roughly equivalent. These findings are reflective of those so often experienced by leukemia cancer patients, and cancer patients in general, in that off target effects of chemotherapeutics on non-cancerous tissues cause significant losses in the functionality of normal cells. This study provides another example of the utility of 3D cell culture systems, particularly in the toxicity testing of pharmaceutical compounds.

## Conclusions

Interaction with bone marrow stromal cells and the extracellular matrix is vital to AML cancer cell survival in that it brings about changes in cell signaling that favor drug resistance and provides a safe haven for leukemia stem cells. Traditional cell propagation methods involve growing cells on flat polystyrene surfaces that have been modified to become hydrophilic and negatively charged when serum containing medium is added which, in turn, allows for cell attachment (86). Growing cells in such 2D cultures presents a host of problems because it does not reflect how cells grow in their natural environment. Inhibitors that are toxic to cells grown in this way have historically suffered significant losses in efficacy when applied to cells grown under more disease-relevant conditions (87). Studies indicate that leukemia cells co-cultured with stromal cells (mostly derived from normal donors) in 3D fair generally better after drug treatment than cells co-cultured in 2D or singly cultured in suspension growth systems. However, while many of these studies use cells of human origin, so far there is a lack of cell systems that use AML and stromal cells from the same patient. Such autologous cell systems would be desirable as stromal cells may also undergo genomic alterations in AML patients. Replicating the AML microenvironment in 3D cultures using patient-specific cell systems in combination with high-throughput synergy screening strategies may offer better hope for identifying combinations of drugs for effective treatment of this devastating disease. Thus, if the 3D leukemia/mesenchymal co-culturing method could be miniaturized and formatted for HTS, more relevant leukemia targeting compounds would likely be identified, and the concept of hindering bone marrow niche-induced chemoresistance may ultimately be realized.

## Author Contributions

All authors listed have made a substantial, direct and intellectual contribution to the work, and approved it for publication.

### Conflict of Interest Statement

The authors declare that the research was conducted in the absence of any commercial or financial relationships that could be construed as a potential conflict of interest.

## Abbreviations

UTR: 3′-untranslated region
ABI1: abl-interactor 1
ALL: acute lymphoblastic leukemia
AF4: acute lymphoblastic leukemia 1-fused gene from chromosome 4
AF6: acute lymphoblastic leukemia 1-fused gene from chromosome 6
AF9: acute lymphoblastic leukemia 1-fused gene from chromosome 9
AF10: acute lymphoblastic leukemia-1 fused gene from chromosome 10
AML: acute myeloid leukemia
AF1p: ALL 1 [acute lymphoblastic leukemia 1]-fused gene from chromosome 1 protein
BCL2/BCL-XL: B-cell lymphoma 2/B-cell lymphoma-extra large
COG: Children's Oncology Group
CAR: chimeric antigen receptor
CLL: chronic lymphoblastic leukemia
CML: chronic myeloid leukemia
CD19: cluster of differentiation 19
CD33: cluster of differentiation 33
CD34: cluster of differentiation 34
c-Myc: c-myelocytomatosis oncogene cellular homolog
CBP: CREB [cAMP (cyclic adenosine monophosphate) response element binding] binding protein
CXCL: cysteine-x-cysteine chemokine ligand
CXCR: cysteine-x-cysteine chemokine receptor
DKK1: Dickkopf-1
DMSO: dimethyl sulfoxide
ELL: eleven-nineteen lysine-rich leukemia
ENL: eleven-nineteen-leukemia
EEN: extra eleven-nineteen leukemia
FLT3: FMS [feline McDonough sarcoma] related tyrosine kinase 3
GAS7: growth arrest specific protein 7
HSCT: hematopoietic stem cell transplantation
HSC: hematopoietic stem cell
HTS: high-throughput screening
HOXA: homeobox A
HOXB: homeobox B
ITD: internal tandem duplications
MTOR: mechanistic target of rapamycin
MLL: mixed lineage leukemia
MDM2: mouse double minute 2 homolog
c-Myb: myeloblastosis viral oncogene homolog
MEIS1: myeloid ecotropic viral integration site 1 homolog
NCI: National Cancer Institute
NSD1: nuclear receptor-binding SET [su(var)3-9, enhancer-of-zeste and trithorax] domain protein 1
NUP98: nucleoporin 98-kDa
PI3K: phosphatidylinositol 3-kinase
PDT: photodynamic therapy
p53: protein 53
P300: protein 300
AKT: protein kinase B
ROS: reactive oxygen species
pRb: retinoblastoma protein
SDT: sonodynamic therapy
starPEG: star-shaped polyethylene glycol
t-AML: therapy related-acute myeloid leukemia
3D: three-dimensional
TSG: tumor suppressor gene
2D: two-dimensional
TKD: tyrosine kinase domain
FDA: US Food and Drug Administration
Wnt: wingless-int.
