# Supplementary material for: Moving Myeloid Leukemia Drug Discovery Into the Third Dimension
Source: Front Pediatr. 2019 Jul 30;7:314. doi: 10.3389/fped.2019.00314 (PMC6682595; doi:10.3389/fped.2019.00314)
Supplement: Supplementary file 1 [file Data_Sheet_1.docx]

**Supplemental Table 1. NCI sponsored clinical trials for leukemias of varying stages and subtypes**

**Number of trials**

**including pediatric /**

**Leukemia type(s) Leukemia stage or subtype** **total trials**

ALL combined; duplicates removed 65 / 130

ALL in remission 19 / 38

ALL recurrent 59 / 118

ALL refractory 34 / 62

CLL combined; duplicates removed 25 / 102

CLL in remission 5 / 7

CLL recurrent 18 / 85

CLL refractory 15 / 77

AML combined; duplicates removed 60 / 230

AML in remission 30 / 57

AML recurrent 50 / 202

AML refractory 21 / 103

CML combined; duplicates removed 23 / 40

CML in remission 7 / 8

CML recurrent 15 / 29

CML refractory 11 / 19

ALL, CLL, AML, CML combined; duplicates removed 109 / 372

**Supplemental Table 2. NCI sponsored clinical trial numbers for ALL, CLL, AML, and CML of varying stages and subtypes**

**Type: ALL**

**Stage or subtype: in remission**

**Includes pediatric:** 2372.00, 2684.00, 14106, 10-050, 2010-0708, 2011-0493, 2012-0999, 2015-0922, 2016-0051, 2016-0137, CASE10Z17, CASE1916, E2993T2, HAPNK1, HSC835, J1055, PBMTC ONC1401, PEDSBMT297, SCT 0208

**Adult:** 9595, 9775, 201303012, 08-087, 12-077, 15-C-0067, 16-1237, 2011-0030, 2014-0844, 2015-0313, 2015-0576, AB-110-001, BMTCTN1101, CASE1Z14, CASE9Z13, IUSCC-0522, J13126, OSU-13219, TK008

**Type: ALL**

**Stage or subtype: recurrent**

**Includes pediatric:** 2546.00, 2684.00, 9716, 10030, 14012, 20150253, 10-050, 11-038, 13-563, 15-384, 15-C-0029, 15-C-0093, 18-C-0059, 2010-0708, 2011-0493, 2012-0999, 2013-1018, 2014-0123, 2014-0521, 2015-0807, 2015-0870, 2016-0211, 2016-0772, AAAK8060, AALL05B1, AALL1621, ACCL15N1CD, ADCT-402-102, ALLR18, CFZ008, CL1-68587-002, CP-103, DCC-3014-01-001, E2993T2, H-31970, HAPNK1, HSC835, J1055, LCCC 1541-ATL, M13-833, M16-106, MAGENTA, MOHEL, PBMTC ONC1401, PEDSBMT295, PEDSBMT297, PEDSCCT5007, PLAT-02, REF2HCT, RELPALL, SCT 0208, SELHEM, STELLA, T2012-002, T2014-001, TSALV, UW13090, 9226, 2016-0137

**Adult:** 2639.00, 9204, 9330, 9364, 9458, 9459, 9775, 13447, 21413, 201303012, J13126, 08-087, 10-C-0054, 11-04-146, 12-077, 14-272, 15-C-0067, 16-1237, 16-1570, 17P.676, 2011-0030, 2013-0116, 2014-0731, 2015-0921, 2016-0402, 2016-0641, 2016-0792, 2016-0973, ADCT-301-002, BCM H 27471, BP-HM-001, CABL001X2101, CASE1Z14, CASE2Z16, CASE9Z13, CCT5001, DS3032-A-U102, DS3201-A-U102, EA9152, I137-102, IIT-2016-RP-HEM-LD-DNR, IMGN632-0801, IRB00007195, IRB14-0709, IRB16-0888, IUSCC-0522, J13126, KTE-C19-103, MCC-14-10739, OSU-13219, OTS167-SE02, OV06-001, PTX-200-AML-015, S1312, SPOC-2010-002, TED10893, TK008, UMCC 2017.056, UPCC 31415

**Type: ALL**

**Stage or subtype: refractory**

**Includes pediatric:** 2684.00, 9226, 9716, 10030, 14106, 20150253, 13-563, 15-384, 15-C-0029, 15-C-0093, 18-C-0059, 2011-0493, 2014-0123, 2014-0521, 2015-0807, 2015-0870, 2016-0051, 2016-0211, 2016-0772, AALL1621, ADCT-402-102, CL1-68587-002, DCC-3014-01-001, HAPNK1, LCCC 1541-ATL, M13-833, M16-106, PEDSCCT5007, PLAT-02, REF2HCT, RELPALL, SELHEM, STELLA, T2014-001

**Adult:** 2639.00, 9364, 9595, 9775, 14-272, 16-1570, 17P.676, 2013-0116, 2015-0313, 2016-0402, 2016-0641, 2016-0792, 2016-0973, 2017-0127, CABL001X2101, CASE2Z16, CASE9Z13, CCT5001, EA9152, IIT-2016-RP-HEM-LD-DNR, IRB00007195, IRB16-0888, IUSCC-0522, KTE-C19-103, PTX-200-AML-015, S1312, TED10893, TK008

**Type: ALL**

**Stage or subtype: combined (in remission, recurrent, refractory); duplicates removed**

**Includes pediatric:** 2372.00, 2546.00, 2684.00, 9226, 9716, 10030, 14012, 14106, 20150253, 10-050, 11-038, 13-563, 15-384, 15-C-0029, 15-C-0093, 18-C-0059, 2010-0708, 2011-0493, 2012-0999, 2013-1018, 2014-0123, 2014-0521, 2015-0807, 2015-0870, 2015-0922, 2016-0051, 2016-0137, 2016-0211, 2016-0772, AAAK8060, AALL05B1, AALL1621, ACCL15N1CD, ADCT-402-102, ALLR18, CASE10Z17, CASE1916, CFZ008, CL1-68587-002, CP-103, DCC-3014-01-001, E2993T2, H-31970, HAPNK1, HSC835, J1055, LCCC 1541-ATL, M13-833, M16-106, MAGENTA, MOHEL, PBMTC ONC1401, PEDSBMT295, PEDSBMT297, PEDSCCT5007, PLAT-02, REF2HCT, RELPALL, SCT 0208, SELHEM, STELLA, T2012-002, T2014-001, TSALV, UW13090

**Adult:** 2639.00, 9204, 9330, 9364, 9458, 9459, 9595, 9775, 13447, 21413, 201303012, J13126, 08-087, 10-C-0054, 11-04-146, 12-077, 14-272, 15-C-0067, 16-1237, 16-1570, 17P.676, 2011-0030, 2013-0116, 2014-0731, 2014-0844, 2015-0313, 2015-0576, 2015-0921, 2016-0402, 2016-0641, 2016-0792, 2016-0973, 2017-0127, AB-110-001, ADCT-301-002, BCM H 27471, BMTCTN1101, BP-HM-001, CABL001X2101, CASE1Z14, CASE2Z16, CASE9Z13, CCT5001, DS3032-A-U102, EA9152, I137-102, IIT-2016-RP-HEM-LD-DNR, IMGN632-0801, IRB00007195, IRB14-0709, IRB16-0888, IUSCC-0522, J13126, KTE-C19-103, MCC-14-10739, OSU-13219, OTS167-SE02, OV06-001, PTX-200-AML-015, S1312, SPOC-2010-002, TED10893, TK008, UMCC 2017.056, UPCC 31415

**Type: CLL**

**Stage or subtype: in remission**

**Includes pediatric:** 2206.00, 10-050, 2015LS034, 2015LS152, MT2005-02

**Adult:** CASE1Z14, INVAC1-CT-201

**Type: CLL**

**Stage or subtype: recurrent**

**Includes pediatric:** 2010.00, 2206.00, 2239.00, 2241.00, 2546.00, 9816, 06-138, 10-050, 2011-0493, 2013-1018, 2015-0807, 2015LS034, 2015LS149, 2015LS152, 2016LS092, DCC-3014-01-001, HSC835, J15165

**Adult:** 2639.00, 9008, 9204, 9738, 13351, 14251, 16009, 151231, 151567, 201303012, 017004, 062-HEM-102, 13-601, 14-396, 15-001217, 15-283, 15-C-0067, 16-1570, 1630GCC, 17-0554, 2014-0115, 2014-0738, 2014-0931, 2015-0860, 2015-1097, 2016-0641, 2016LS107, 3475-155, AAAP5661, ACE-CL-003, ARQ 531-101, ASN002-101, C34001, CA224-022, CASE1Z14, CASE2Z16, CASE9Z13, CC-122-CLL-001, CIRM-0001, D17093, DCL-16-001, GO29781, GS-US-401-1757, HEM0048, I137-102, IMMU-114-01, IPI-145-12, IRB17-0901, MC128A, MC1485, MCC-14-10739, ME-401-002, ME-522-001, MOR208C205, MT-3724_NHL_001, OSU-14087, Pro00086177, S17-00042, STUDY00017005, TED10893, TG-UPCC-108, U2-VEN-109, UC-961, UMCC 2012.025, UPCC 18415, UW14113, VICC BMT 1651

**Type: CLL**

**Stage or subtype: refractory**

**Includes pediatric:** 2206.00, 2239.00, 2241.00, 06-138, 2011-0493, 2015-0807, 2015LS149, 2016LS132, CHARKALL, DCC-3014-01-001, H-31970, J1055, MT1999-14, MT2005-02, MT2005-10

**Adult:** GS-US-401-1757, 2639.00, 9008, 9330, 14251, 16009, 151567, VICC BMT 1651, 017004, 062-HEM-102, 09-421, 13-601, 14-396, 15-283, 15-C-0067, 16-1570, 1630GCC, 17-0554, 2014-0115, 2014-0931, 2015-0860, 2015-1097, 2016-0641, 2016LS107, 3475-155, AAAP5661, ACE-CL-003, ARQ 531-101, ASN002-101, BCM H 27471, C34001, CA224-022, CASE1Z14, CASE2Z16, CASE9Z13, CC-122-CLL-001, D17093, D6910C00001, DCL-16-001, GO29781, HEM0048, IMMU-114-01, IPI-145-12, IRB17-0901, MC1485, ME-401-002, ME-522-001, MOR208C205, MRG106-11-101, MT-3724_NHL_001, OSU-14087, P1446A-05/79/13, PCYC-1103-CA, Pro00086177, R1979-HM-1333, SPOC-2010-002, STUDY00017005, TED10893, TG-UPCC-108, U2-VEN-109, UC-961, UW14113

**Type: CLL**

**Stage or subtype: combined (in remission, recurrent, refractory); duplicates removed**

**Includes pediatric:** 2010.00, 2206.00, 2239.00, 2241.00, 2546.00, 9816, 06-138, 10-050, 2011-0493, 2013-1018, 2015-0807, 2015LS034, 2015LS149, 2015LS152, 2016LS092, 2016LS132, CHARKALL, DCC-3014-01-001, H-31970, HSC835, J1055, J15165, MT1999-14, MT2005-02, MT2005-10

**Adult:** 2639.00, 9008, 9204, 9330, 9738, 13351, 14251, 16009, 151231, 151567, 201303012, 017004, 062-HEM-102, 09-421, 13-601, 14-396, 15-001217, 15-283, 15-C-0067, 16-1570, 1630GCC, 17-0554, 2014-0115, 2014-0738, 2014-0931, 2015-0860, 2015-1097, 2016-0641, 2016LS107, 3475-155, AAAP5661, ACE-CL-003, ARQ 531-101, ASN002-101, BCM H 27471, C34001, CA224-022, CASE1Z14, CASE2Z16, CASE9Z13, CC-122-CLL-001, CIRM-0001, D17093, D6910C00001, DCL-16-001, GO29781, GS-US-401-1757, HEM0048, I137-102, IMMU-114-01, INVAC1-CT-201, IPI-145-12, IRB17-0901, MC128A, MC1485, MCC-14-10739, ME-401-002, ME-522-001, MOR208C205, MRG106-11-101, MT-3724_NHL_001, OSU-14087, P1446A-05/79/13, PCYC-1103-CA, Pro00086177, R1979-HM-1333, S17-00042, SPOC-2010-002, STUDY00017005, TED10893, TG-UPCC-108, U2-VEN-109, UC-961, UMCC 2012.025, UPCC 18415, UW14113, VICC BMT 1651

**Type: AML**

**Stage or subtype: in remission**

**Includes pediatric:** 2010.00, 2372.00, 2684.00, 13272, 14106, 10-050, 12-053, 13-192, 15D.323, 2011-0493, 2012-0708, 2012-0819, 2012-0999, 2014-0431, 2015LS152, 2016-0137, 9L-11-8, CASE10Z17, HAPNK1, HCI61077, HSC835, J1055, MT2005-02, MT2005-10, MT2006-13, PBMTC ONC1401, SCT 0208, SCT 0307, UCAML, UPCI 13-131

**Adult:** 9595, 11702, 17222, 201303012, 201401085, 201606003, 08-087, 12-077, 12-1558, 16-1237, 17-639, 2014-0116, 2014-0738, 2015-0213, 2215-CL-0302, AB-110-001, CASE1Z14, CASE9Z13, E1900T2, IUSCC-0522, J1240, J13126, OSU-13219, Pro00091220, S0106A, STML-401-0214, UCDCC#227

**Type: AML**

**Stage or subtype: recurrent**

**Includes pediatric:** 2010.00, 2498.00, 2546.00, 2684.00, 9226, 9716, 13272, 14012, 201709041, 10-050, 12-053, 12D.501, 13-563, 15-384, 15-C-0093, 16-6007, 17-327, 2011-0493, 2012-0708, 2012-0819, 2012-0999, 2015-0807, 2016-0137, 2016-0341, 2016-0772, 9L-11-8, AAAK8060, AAML10B14, AAML1421, ADSPAM, CASE10Z17, DCC-3014-01-001, HAPNK1, HCI61077, HSC835, J1055, M13-833, MOHEL, MT2005-10, PANAML, PBMTC ONC1401, PEDSBMT295, REF2HCT, SCT 0208, SCT 0307, SELHEM, T2016-003, UCAML, UW13090, VENAML

**Adult:** 1315.2, 9204, 9567, 9595, 9713, 10026, 10075, 14269, 16270, 19036, 116183, 20120252, 20160377, 201303012, 201401085, 201610088, 201703089, 1507016193, J13126, 08-087, 1101-HEM-101, 12D.587, 14-213, 14-222, 14-272, 1565GCC, 15-C-0067, 15D.377, 16-464, 16-574, 16C.773, 17-056, 17-0754, 17-099, 2012-0079, 2012-0648, 2012-1047, 2013-0116, 2014-0057, 2014-0344, 2014-0358, 2014-0731, 2014-0861, 2014-0907, 2014-0975, 2015-0313, 2015-0436, 2015-0665, 2016-0250, 2016-0444, 2016-0889, 2016-0979, 2016LS056, 2016LS153, 2017-0337, 2017-0490, 2017-0912, 2102-HEM-101, 5F9005, 9L-16-3, 9L-16-6, AAAQ8444, ADCT-301-002, AGS62P1-16-1, AGS67E-15-2, ALRN-6924-1-02, AMV564-101, ARO-010, ARO-013, BBI-DSP7888-101, BGBC003, BP-011, BP-HM-001, BS FF1050101US01, C34002, CASE1917, CASE1Z14, CASE2Z16, CASE9Z13, CC #139510, CC-90002-AML-001, CC-90009-AML-001, CCCWFU # 22A15, CCCWFU 22214, CCCWFU 22215, CCCWFU#99213, CL1-64315-001, CLGH447X2102, CPDR001X2105, CP-MGD006-01, CR108147, D8540C00001, DS3032-A-U102, DS3201-A-U102, E1900T2, FF-10101-US101/201, GO30139, GO39902, GS-US-339-1559, HEMAML0023, HM-091, I137-102, IIT-2016-RP-HEM-LD-DNR, IMGN632-0801, IMGN779 0601, INCB 53914-101, IRB00007195, IRB14-0709, IRB16-0888, IRB16-1195, IUSCC-0522, J1240, J15219, J1651, JW-231A-103, LAM-003-HEM-CLN02, LCCC1522, M15-913, M16-183, M16-186, M16-415, MB-104, MC1488, MC1684, MCC-14-10739, NU 17H04, ONO-7475-01, ORH2014-001, OSU-13219, OSU-14169, OTS167-SE02, OV06-001, OX1222, PLX122-01, PTX-200-AML-015, RT12-US-AML-a, S9031-S9126-S9333-S9500-B, SCI-CD47-002, SGI-110-06, STML-401-0114, STUDY00017583, SY-1425-201, TED10893, TK008, TPI-ALV-201, TROV-052, UCART123_01, UMCC 2014.107, UMCC 2017.056, UPCC 04415, WO29519, XmAb14045-01

**Type: AML**

**Stage or subtype: refractory**

**Includes pediatric:** 2684.00, 9226, 9716, 13272, 14106, 12D.501, 13-563, 15-384, 15-C-0093, 16-6007, 17-327, 2016-0341, 2016-0772, ADSPAM, BMT236, DCC-3014-01-001, HCI61077, M13-833, REF2HCT, T2016-003, VENAML

**Adult:** 1315.2, 9567, 9713, 10026, 10075, 14269, 16270, 20160377, 201401085, 201610088, 201703089, 1101-HEM-101, 12D.587, 14-222, 15D.377, 16-464, 16-574, 16C.773, 17-056, 17-0754, 17-099, 2012-0079, 2012-0648, 2012-1047, 2013-0116, 2015-0436, 2015-0665, 2016-0250, 2016-0444, 2016-0889, 2016-0979, 2016LS056, 2016LS153, 2017-0337, 2017-0490, 2017-0912, 2102-HEM-101, 5F9005, 9L-16-6, AGS62P1-16-1, ALRN-6924-1-02, AMV564-101, ARO-013, BP-011, C34002, CASE1917, CASE2Z16, CCCWFU 22214, CCCWFU 22215, CL1-64315-001, CPDR001X2105, CR108147, D8540C00001, FF-10101-US101/201, GO39902, GS-US-339-1559, HM-091, IMGN632-0801, IMGN779 0601, INCB 53914-101, IUSCC-0522, J1651, LCCC1522, M14-546, M15-913, M16-183, M16-186, M16-415, MB-104, MC1488, MC1684, NU 17H04, ONO-7475-01, OSU-14169, SCI-CD47-002, STUDY00017583, SY-1425-201, TED10893, TPI-ALV-201, TROV-052, UCART123_01, WO29519

**Type: AML**

**Stage or subtype: combined (in remission, recurrent, refractory); duplicates removed**

**Includes pediatric:** 2010.00, 2372.00, 2498.00, 2546.00, 2684.00, 9226, 9716, 13272, 14012, 14106, 201709041, 10-050, 12-053, 12D.501, 13-192, 13-563, 15-384, 15-C-0093, 15D.323, 16-6007, 17-327, 2011-0493, 2012-0708, 2012-0819, 2012-0999, 2014-0431, 2015-0807, 2015LS152, 2016-0137, 2016-0341, 2016-0772, 9L-11-8, AAAK8060, AAML10B14, AAML1421, ADSPAM, BMT236, CASE10Z17, DCC-3014-01-001, HAPNK1, HCI61077, HSC835, J1055, M13-833, MOHEL, MT2005-02, MT2005-10, MT2006-13, PANAML, PBMTC ONC1401, PEDSBMT295, REF2HCT, SCT 0208, SCT 0307, SELHEM, T2016-003, UCAML, UPCI 13-131, UW13090, VENAML

**Adult:** 1315.2, 9204, 9567, 9595, 9713, 10026, 10075, 11702, 14269, 16270, 17222, 19036, 116183, 20120252, 20160377, 201303012, 201401085, 201606003, 201610088, 201703089, 1507016193, J13126, 08-087, 1101-HEM-101, 12-077, 12-1558, 12D.587, 14-213, 14-222, 14-272, 1565GCC, 15-C-0067, 15D.377, 16-1237, 16-464, 16-574, 16C.773, 17-056, 17-0754, 17-099, 17-639, 2012-0079, 2012-0648, 2012-1047, 2013-0116, 2014-0057, 2014-0116, 2014-0344, 2014-0358, 2014-0731, 2014-0738, 2014-0861, 2014-0907, 2014-0975, 2015-0213, 2015-0313, 2015-0436, 2015-0665, 2016-0250, 2016-0444, 2016-0889, 2016-0979, 2016LS056, 2016LS153, 2017-0337, 2017-0490, 2017-0912, 2102-HEM-101, 2215-CL-0302, 5F9005, 9L-16-3, 9L-16-6, AAAQ8444, AB-110-001, ADCT-301-002, AGS62P1-16-1, AGS67E-15-2, ALRN-6924-1-02, AMV564-101, ARO-010, ARO-013, BBI-DSP7888-101, BGBC003, BP-011, BP-HM-001, BS FF1050101US01, C34002, CASE1917, CASE1Z14, CASE2Z16, CASE9Z13, CC #139510, CC-90002-AML-001, CC-90009-AML-001, CCCWFU # 22A15, CCCWFU 22214, CCCWFU 22215, CCCWFU#99213, CL1-64315-001, CLGH447X2102, CPDR001X2105, CP-MGD006-01, CR108147, D8540C00001, DS3032-A-U102, DS3201-A-U102, E1900T2, FF-10101-US101/201, GO30139, GO39902, GS-US-339-1559, HEMAML0023, HM-091, I137-102, IIT-2016-RP-HEM-LD-DNR, IMGN632-0801, IMGN779 0601, INCB 53914-101, IRB00007195, IRB14-0709, IRB16-0888, IRB16-1195, IUSCC-0522, J1240, J13126, J15219, J1651, JW-231A-103, LAM-003-HEM-CLN02, LCCC1522, M14-546, M15-913, M16-183, M16-186, M16-415, MB-104, MC1488, MC1684, MCC-14-10739, NU 17H04, ONO-7475-01, ORH2014-001, OSU-13219, OSU-14169, OTS167-SE02, OV06-001, OX1222, PLX122-01, Pro00091220, PTX-200-AML-015, RT12-US-AML-a, S0106A, S9031-S9126-S9333-S9500-B, SCI-CD47-002, SGI-110-06, STML-401-0114, STML-401-0214, STUDY00017583, SY-1425-201, TED10893, TK008, TPI-ALV-201, TROV-052, UCART123_01, UCDCC#227, UMCC 2014.107, UMCC 2017.056, UPCC 04415, WO29519, XmAb14045-01

**Type: CML**

**Stage or subtype: in remission**

**Includes pediatric:** 2206.00, 2012-0708, 2015LS034, 2015LS152, 2016-0137, I 40916, MT2005-02

**Adult:** CASE1Z14

**Type: CML**

**Stage or subtype: recurrent**

**Includes pediatric:** 2010.00, 2546.00, 2684.00, 9816, 13-563, 18-C-0059, 2012-0708, 2012-0819, 2015LS152, 2016-0137, CASE1916, PEDSBMT295, REF2HCT, SCT 0208, Winship3121-15

**Adult:** 9204, 201303012, 08-087, 12-077, 14-272, 15-C-0067, 2012-0648, 2014-0435, 2016LS107, CABL001X2101, CASE2Z16, MCC-14-10739, PTX-200-AML-015, XmAb14045-01

**Type: CML**

**Stage or subtype: refractory**

**Includes pediatric:** 2239.00, 13-563, 18-C-0059, 2012-0819, CASE1916, HAPNK1, HSC835, J15165, PEDSBMT295, REF2HCT, SCT 0208

**Adult:** 2012-0648, 2014-0435, AP24534-14-203, CABL001X2101, CASE2Z16, PTX-200-AML-015, S17-00042, XmAb14045-01

**Type: CML**

**Stage or subtype: combined (in remission, recurrent, refractory); duplicates removed**

**Includes pediatric:** 2010.00, 2206.00, 2239.00, 2546.00, 2684.00, 9816, 13-563, 18-C-0059, 2012-0708, 2012-0819, 2015LS034, 2015LS152, 2016-0137, CASE1916, HAPNK1, HSC835, I 40916, J15165, MT2005-02, PEDSBMT295, REF2HCT, SCT 0208, Winship3121-15

**Adult:** 9204, 201303012, 08-087, 12-077, 14-272, 15-C-0067, 2012-0648, 2014-0435, 2016LS107, AP24534-14-203, CABL001X2101, CASE1Z14, CASE2Z16, MCC-14-10739, PTX-200-AML-015, S17-00042, XmAb14045-01

**Type: ALL, CLL, AML, CML**

**Stage or subtype: combined (in remission, recurrent, refractory); duplicates removed**

**Includes pediatric:** 2010.00, 2206.00, 2239.00, 2241.00, 2372.00, 2498.00, 2546.00, 2684.00, 9226, 9716, 9816, 10030, 13272, 14012, 14106, 20150253, 201709041, 06-138, 10-050, 11-038, 12-053, 12D.501, 13-192, 13-563, 15-384, 15-C-0029, 15-C-0093, 15D.323, 16-6007, 17-327, 18-C-0059, 2010-0708, 2011-0493, 2012-0708, 2012-0819, 2012-0999, 2013-1018, 2014-0123, 2014-0431, 2014-0521, 2015-0807, 2015-0870, 2015-0922, 2015LS034, 2015LS149, 2015LS152, 2016-0051, 2016-0137, 2016-0211, 2016-0341, 2016-0772, 2016LS092, 2016LS132, 9L-11-8, AAAK8060, AALL05B1, AALL1621, AAML10B14, AAML1421, ACCL15N1CD, ADCT-402-102, ADSPAM, ALLR18, BMT236, CASE10Z17, CASE1916, CFZ008, CHARKALL, CL1-68587-002, CP-103, DCC-3014-01-001, E2993T2, H-31970, HAPNK1, HCI61077, HSC835, I 40916, J1055, J15165, LCCC 1541-ATL, M13-833, M16-106, MAGENTA, MOHEL, MT1999-14, MT2005-02, MT2005-10, MT2006-13, PANAML, PBMTC ONC1401, PEDSBMT295, PEDSBMT297, PEDSCCT5007, PLAT-02, REF2HCT, RELPALL, SCT 0208, SCT 0307, SELHEM, STELLA, T2012-002, T2014-001, T2016-003, TSALV, UCAML, UPCI 13-131, UW13090, VENAML, Winship3121-15

**Adult:** 1315.2, 2639.00, 9008, 9204, 9330, 9364, 9458, 9459, 9567, 9595, 9713, 9738, 9775, 10026, 10075, 11702, 13351, 13447, 14251, 14269, 16009, 16270, 17222, 19036, 21413, 116183, 151231, 151567, 20120252, 20160377, 201303012, 201401085, 201606003, 201610088, 201703089, 1507016193, J13126, 017004, 062-HEM-102, 08-087, 09-421, 10-C-0054, 1101-HEM-101, 11-04-146, 12-077, 12-1558, 12D.587, 13-601, 14-213, 14-222, 14-272, 14-396, 15-001217, 15-283, 1565GCC, 15-C-0067, 15D.377, 16-1237, 16-1570, 1630GCC, 16-464, 16-574, 16C.773, 17-0554, 17-056, 17-0754, 17-099, 17-639, 17P.676, 2011-0030, 2012-0079, 2012-0648, 2012-1047, 2013-0116, 2014-0057, 2014-0115, 2014-0116, 2014-0344, 2014-0358, 2014-0435, 2014-0731, 2014-0738, 2014-0844, 2014-0861, 2014-0907, 2014-0931, 2014-0975, 2015-0213, 2015-0313, 2015-0436, 2015-0576, 2015-0665, 2015-0860, 2015-0921, 2015-1097, 2016-0250, 2016-0402, 2016-0444, 2016-0641, 2016-0792, 2016-0889, 2016-0973, 2016-0979, 2016LS056, 2016LS107, 2016LS153, 2017-0127, 2017-0337, 2017-0490, 2017-0912, 2102-HEM-101, 2215-CL-0302, 3475-155, 5F9005, 9L-16-3, 9L-16-6, AAAP5661, AAAQ8444, AB-110-001, ACE-CL-003, ADCT-301-002, AGS62P1-16-1, AGS67E-15-2, ALRN-6924-1-02, AMV564-101, AP24534-14-203, ARO-010, ARO-013, ARQ 531-101, ASN002-101, BBI-DSP7888-101, BCM H 27471, BGBC003, BMTCTN1101, BP-011, BP-HM-001, BS FF1050101US01, C34001, C34002, CA224-022, CABL001X2101, CASE1917, CASE1Z14, CASE2Z16, CASE9Z13, CC #139510, CC-122-CLL-001, CC-90002-AML-001, CC-90009-AML-001, CCCWFU # 22A15, CCCWFU 22214, CCCWFU 22215, CCCWFU#99213, CCT5001, CIRM-0001, CL1-64315-001, CLGH447X2102, CPDR001X2105, CP-MGD006-01, CR108147, D17093, D6910C00001, D8540C00001, DCL-16-001, DS3032-A-U102, DS3201-A-U102, E1900T2, EA9152, FF-10101-US101/201, GO29781, GO30139, GO39902, GS-US-339-1559, GS-US-401-1757, HEM0048, HEMAML0023, HM-091, I137-102, IIT-2016-RP-HEM-LD-DNR, IMGN632-0801, IMGN779 0601, IMMU-114-01, INCB 53914-101, INVAC1-CT-201, IPI-145-12, IRB00007195, IRB14-0709, IRB16-0888, IRB16-1195, IRB17-0901, IUSCC-0522, J1240, J13126, J15219, J1651, JW-231A-103, KTE-C19-103, LAM-003-HEM-CLN02, LCCC1522, M14-546, M15-913, M16-183, M16-186, M16-415, MB-104, MC128A, MC1485, MC1488, MC1684, MCC-14-10739, ME-401-002, ME-522-001, MOR208C205, MRG106-11-101, MT-3724_NHL_001, NU 17H04, ONO-7475-01, ORH2014-001, OSU-13219, OSU-14087, OSU-14169, OTS167-SE02, OV06-001, OX1222, P1446A-05/79/13, PCYC-1103-CA, PLX122-01, Pro00086177, Pro00091220, PTX-200-AML-015, R1979-HM-1333, RT12-US-AML-a, S0106A, S1312, S17-00042, S9031-S9126-S9333-S9500-B, SCI-CD47-002, SGI-110-06, SPOC-2010-002, STML-401-0114, STML-401-0214, STUDY00017005, STUDY00017583, SY-1425-201, TED10893, TG-UPCC-108, TK008, TPI-ALV-201, TROV-052, U2-VEN-109, UC-961, UCART123_01, UCDCC#227, UMCC 2012.025, UMCC 2014.107, UMCC 2017.056, UPCC 04415, UPCC 18415, UPCC 31415, UW14113, VICC BMT 1651, WO29519, XmAb14045-01

**Supplemental Table 3. COG sponsored clinical trials for childhood leukemias**

**Leukemia type** **COG Number**

ALL ACCL1033

ALL, newly diagnosed ACCL1333

ALL, Philadelphia Chromosome-Positive (Ph+) AALL1631

ALL and Lysine Methyltransferase 2A Mixed Lineage Leukemia

(*KMT2A MLL*) gene rearrangement AALL15P1

ALL with Cytokine Receptor-Like Factor 2-Rearranged (*CRLF2-R*)

and/or Janus Kinase (JAK) pathway mutation AALL1521

B-ALL ANHL1131

B-ALL, newly diagnosed AALL0932

B-ALL, first relapse AALL1331

B-ALL that is CD22+ and is of relapsed or refractory stage or subtype AALL1621

B-precursor ALL, including Ph-like Tyrosine Kinase Inhibitor (TKI)

sensitive mutations AALL1131

T-ALL, newly diagnosed AALL1231

ALL or AML ACCL0934

AML ACCL0933

AML, newly diagnosed AAML0531

AML, relapsed AAML1421

AML with high allelic ratio FMS [Feline McDonough Sarcoma] Related

Tyrosine Kinase 3/Internal Tandem Duplications (FLT3/ITD) AAML1031

AML, Down Syndrome AAML1531

Acute Promyelocytic Leukemia (APL), newly diagnosed AAML1331
